# Development and Validation of the Reasons to Exergame (RTEX) Scale in Young Adults: Exploratory Factors Analysis

**DOI:** 10.2196/16261

**Published:** 2020-06-15

**Authors:** Erin O'Loughlin, Catherine M Sabiston, Lisa Kakinami, Jennifer J McGrath, Mia Consalvo, Jennifer L O'Loughlin, Tracie A Barnett

**Affiliations:** 1 Centre de Recherche du CHUM Concordia University Montreal, QC Canada; 2 Department of Social and Preventive Medicine Université de Montréal Montreal, QC Canada; 3 Individualized Program Department Concordia University Montreal, QC Canada; 4 Division of Neurosurgery Faculty of Kinesiology and Physical Education University of Toronto Toronto, ON Canada; 5 Department of Family Medicine McGill University Montreal, QC Canada; 6 PERFORM Centre Concordia University Montreal, QC Canada; 7 Psychology Department Concordia University Montreal, QC Canada; 8 Sainte-Justine Research Centre Université de Montréal Montreal, QC Canada; 9 Epidemiology and Biostatistics Unit Centre Armand-Frappier Santé Biotechnologie Institut National de la Recherche Scientifique Laval, QC Canada

**Keywords:** exergaming, youth, young adults, motivation, scale

## Abstract

**Background:**

Exergaming is associated with positive health benefits; however, little is known about what motivates young people to exergame.

**Objective:**

This study aimed to develop a new Reasons to Exergame (RTEX) scale and describe its psychometric properties (Study 1) including test-retest reliability (Study 2). We also examined the test-retest reliability of self-report exergaming behavior measures (Study 2).

**Methods:**

We identified scale items in consultation with experts. In Study 1, we conducted an Exploratory Factor Analysis of RTEX and examined how the factors identified relate to exergaming frequency and intensity in a population-based sample of 272 young adults. In Study 2, we examined the test-retest reliability of RTEX factors and self-report measures of past-week exergaming frequency and intensity among 147 college students.

**Results:**

We identified four factors in RTEX: exergaming for fitness, exergaming for enjoyment, preferring exergaming over other gaming options, and choosing exergaming over competing interests (eg, sports). Test-retest reliability of RTEX factors (ICC 0.7-0.8) and self-report exergaming frequency (ICC 0.4-0.9) was adequate. Exergaming for fitness and enjoyment were positively associated with the frequency of exergaming with friends and family, and with exergaming intensity. Preferring exergaming over other gaming options and choosing exergaming over competing interests (eg, sports) were not related to exergaming behavior.

**Conclusions:**

RTEX is a psychometrically sound scale with four factors that measure reasons to exergame. Replication of these findings is needed in larger, more diverse samples.

## Introduction

Physically active lifestyles should be promoted early in life because fewer than 60% of youth meet the recommended physical activity (PA) levels and because PA levels established in childhood track into adulthood [1-4]. However, both physical inactivity and sedentary behavior are increasing among youths [5,6], in part due to the popularity of screen activities such as television viewing and video game playing (ie, gaming). An estimated 97% of Americans aged 12-17 years [7] and 56% of young adults aged 18-25 years report past-year gaming using personal computers, laptops, consoles, or smartphones [8]. The average time spent gaming among youths has increased, and young adults in 2018 spend more than 7 hours weekly playing video games [9].

There is, however, a type of gaming that may confer physical and mental health benefits and that does not contribute to sedentary behavior. Active video game-playing, also called “exergaming,” is a contemporary alternative to traditional gaming that incorporates PA into the video game concept. Exergaming increases energy expenditure [10-15] and PA [16-18], helps players manage weight [19-21], improves mental health [22], and can replace sedentary behavior [18,22-24]. However, both anecdotal and qualitative evidence suggest that, in contrast to more sedentary gaming [25,26], motivation for exergaming can decrease over time as novelty diminishes or because of competing interests. Technical difficulties in exergaming (eg, console malfunctioning, over- or undersensitivity of controls, inaccurate tracking) [18,27,28], insufficient design features of gaming (eg, lack of compelling narratives), or PA-related discomfort during exergaming (eg, sweating, breathing hard while playing) could also contribute to declines. Similar to both traditional gaming and PA in youths (eg, males initiate gaming at a younger age than females; PA declines much earlier and more rapidly in females than males), these factors may differ by gender [29-32].

Because of the health-enhancing potential of exergaming, it is key to better understand the reasons why young people choose to exergame and maintain this behavior. Previous studies have examined exergaming motivation using scales that were not developed specifically for exergaming, such as those targeting general PA [33]. Fitzgerald et al [34] used the Self-Motivation Inventory (a 40-item questionnaire that assesses the trait of self-motivation, such as the inherent ability to persevere at a task) and the Intrinsic Motivation Inventory (a questionnaire based on Self-Determination Theory) to assess motivation for exergaming. Sun et al [35] applied the 15-item Situational Interest Scale - Elementary School to exergaming among physical education students in elementary school. However, exergaming may not be viewed as PA by young people, and these scales might not distinguish between motivation for the PA aspects of exergaming and motivation for the gaming aspects.

In the only study to date that examined motivation for exergaming using a scale specifically developed for exergaming, Staino et al [36,37] used the self-report Motivation for Exergame Play Inventory, a validated 28-item questionnaire that assesses motivation for exergaming among youths with overweight or obesity. However, the Motivation for Exergame Play Inventory measures motivation after an acute bout of exergaming related to the specific game just played, not motivation toward exergaming in general, and is therefore not ideal for examining exergaming motivation in research and surveillance. Although increased understanding of the reasons that young people choose to exergame could improve exergaming interventions as well as public health messaging about exergaming, an important gap in the literature is the lack of evidence on reasons to initiate and sustain exergaming.

The purpose of this study was to develop a new Reasons to Exergame (RTEX) scale that is useful in intervention studies, observational studies, and surveillance and to test its psychometric properties. Although assessment of reasons to exergame is also important in children and older adults, this first examination of RTEX was undertaken in young adults who are technologically sophisticated, often highly engaged in gaming, and in whom PA levels tend to decline due to competing priorities, lack of time, and lack of motivation.

After item generation for RTEX, two studies were conducted, each using different databases. Study 1 employed data from the Nicotine Dependence in Teens (NDIT) Study, a longitudinal investigation of youths aged 12-13 years at inception with follow-up to age 30 years [38]. In Study 1, we conducted an Exploratory Factor Analysis (EFA) of RTEX items and studied how factors identified in the EFA relate to the frequency and intensity of exergaming to test convergent validity. Because the NDIT data collection time points were fixed, it was not possible to collect test-retest data within the NDIT protocol. Therefore, Study 2 was undertaken to assess the test-retest reliability of both the RTEX scale and self-report measures of exergaming frequency and intensity. Study 2 used a convenience sample of college students. Methods and results are presented first for Study 1 and then for Study 2.

## Methods

### Study 1

Data for Study 1 were collected in self-report questionnaires completed in 2017-2019 by 630 young adults participating in the NDIT Study [38], an ongoing investigation of grade 7 students recruited in 10 Montreal-area high schools in 1999-2000. Of 2325 eligible students, 56% participated at baseline. The low response was attributable to the need for blood samples for genotyping and a labor dispute in Quebec that resulted in numerous teachers refusing to collect consent forms. No data were collected from nonrespondents [38]. Self-report questionnaires were administered at school every 3 months during the 10-month school year in grades 7-11, for a total of 20 cycles during the 5 years of high school. Post high school data were collected in self-report questionnaires in 2007-2008 (cycle 21), 2011-2012 (cycle 22), and 2017-2019 (cycle 23) when participants were aged 20, 24, and 30 years, on average, respectively. This study used data from cycle 23. The NDIT Study was approved by the Ethics Committee of the Centre de Recherche du Centre Hospitalier de l’Université de Montréal (ND 06.087).

#### Measures

##### RTEX

Items for the RTEX scale were selected by the authors in consultation with PA and exergaming experts and with reference to constructs relevant to exergaming such as general interest in gaming, social gaming preferences, degree of enjoyment in exergaming, reasons to be physically active (eg, lose weight, increase strength), and comparing exergaming to other active pastimes (such as sports). Items drew on the Gaming Motivation Scale [39], the Intrinsic Motivations to Gameplay scale [40], and the Exercise Motivations Inventory-2 [41]. Experts judged redundancy, applicability, and representativeness of the items in an exergaming context. The scale was pilot-tested among experts (PA and exergaming researchers) as well as among young adults during development of the NDIT cycle 23 questionnaire for clarity of wording, item choice, and clarity of response choices. Participants were asked to indicate how true each item was regarding their reasons to exergame using a 5-point Likert-type response format (ie, completely false, slightly false, neither true nor false, slightly true, completely true).

##### Frequency and Intensity of Exergaming

Items measuring exergaming behavior were modeled on the short-form self-administered usual-week International Physical Activity Questionnaire, which is used in cross-national monitoring of PA in youths and adults and demonstrates reliability and validity against the accelerometer [42]. Specifically, participants were asked, “Do you exergame using consoles such as Nintendo Wii, XBOX ONE Kinect, Sony Play Station Move, Sony Eye Toy: Kinetic, or using your cellphone and/or a mobile app? (ex. ZOMBIES, RUN! Nike+ Running App, Fit Freeway, Pokémon Go).” Response options were (yes and no). Those who responded “yes” were asked how many days per week they played active video games (or exergamed; options: 1-7 days); how many minutes (on average) they played each time (open-ended); and perceived effort of play (light, moderate, vigorous). All three measures were used in both studies. Finally, we assessed how frequently participants exergamed alone, with friends, or with family by asking, “How often do you exergame with… (friends, family, alone)?” Response options included never, rarely, sometimes, often, and very often. This measure was used only in Study 1.

#### Data Analysis

##### Exploratory Factor Analysis

Use of EFA at this phase in the development of the RTEX is appropriate, given that the items are new and there was uncertainty in how the items would load on the factors [43]. Since the 26 possible items for RTEX were expected to intercorrelate, they were subjected to an EFA using maximum likelihood and oblique promax rotation (direct oblimin) for factor derivation. Criteria [44-49] used to establish the number of factors to retain were the Cattell scree plot, >10% of variance accounted for by each factor, pattern coefficients of >0.40 on a given factor [46,47], interpretability of the factors, and internal reliability (Cronbach α) of >0.7 for each factor [44]. The Kaiser-Meyer-Olkin test was used to measure sampling adequacy for conducting a factor analysis. Factor items were determined using eigenvalues >1.

As indicated, 26 items were retained in RTEX for the initial EFA. The Kaiser-Meyer-Olkin measure of sampling adequacy (0.90) indicated adequate sample size for the analysis, and the Bartlett test of sphericity (4494.7_325_, *P*<.001) indicated that the correlation matrix was appropriate for this analysis. Six factors with eigenvalues >1.0 [48] were extracted from the matrix, explaining 71.2% of the variance. Based on the analysis of the loadings of the rotated factors (the pattern matrix), three items were dropped from the item pool because they failed to load >0.40 on any of the 4 factors (Multimedia Appendix 1). We repeated the factor analysis with the remaining 23 items and examined the factor loadings of the new promax-rotated factor solution. Five factors emerged, which explained 70.0% of the total variance. Inspection of the pattern matrix showed that all items but one loaded >0.40 on one of the 5 factors (Multimedia Appendix 1), and none of the items had high cross-loading on other factors. Therefore, after removing the single item, the process was repeated a final time, and a 4-factor solution explaining 66% of the variance was retained (n=22 items). Factor scores were calculated by summing the individual items within a factor and averaging the score by the number of items with responses to create one score per factor (see Results section).

##### Convergent Validity

The associations between each RTEX factor and exergaming frequency (minutes per week) and intensity (light, moderate, intense) were examined in separate multivariable linear regression models. We also investigated the association between each RTEX factor and the frequency of exergaming alone, with friends, and with family, in three separate multivariable linear regression models. All regression models were controlled for age, sex, mother attended university (yes/no), and the other RTEX factors. Sex differences in RTEX factors and individual items were examined using independent *t* tests.

Students (54.6% female; mean age 19.5, SD 0.5) recruited in an Exercise Science Department in a large urban university (n=147) completed the RTEX on two occasions (Time 1 and Time 2) 7-9 days apart in winter 2017.

The study received approval from the ethics and protection review boards of Concordia University (30007966), and the students provided assent during questionnaire administration. Students received class credit for completing the study.

### Study 2

#### Data Analysis

Intraclass correlations (ICCs) were used to examine test-retest reliability between responses over time for continuous variables including exergaming intensity and days per week exergaming Two-way random ICCs were computed and the average ICC was reported [49]. Adequate test-retest reliability was defined as ICC≥0.75 [11]. The weighted Kappa statistic (κ) was used to describe the test-retest reliability of categorical variables (such as ever exergamed, yes/no), and the strength of agreement between responses was defined as poor to fair (κ=0.0-0.4), moderate (κ=0.41-0.6), substantial (κ=0.61-0.8), and almost perfect (κ=0.81-1.0). Statistical significance was set at 0.05.

## Results

### Study 1

Table 1 shows selected characteristics of NDIT participants (N=272) who responded yes to ever exergaming and were therefore retained in the analytic sample.

**Table 1:** Selected characteristics of participants in Study 1 (N=272), NDIT 2017-2019.

Characteristic		Value
Age (years), mean (SD)		30.2 (0.7)
Male, n (%)		127 (46.8)
French-speaking, n (%)		98 (36.0)
Mother university-educated, n (%)		109 (40.2)
Canadian-born, n (%)		262 (96.4)
**Minutes exergaming/week, mean (SD)**		
	All participants	30.1 (90.0)
	Excluding those reporting 0 minutes	147.0 (150.7)
**Exergaming intensity, n (%)**		
	Light	176 (64.8)
	Moderate	92 (33.8)
	Intense	4 (1.4)
**Frequency of exergaming, mean (SD)**		
	Alone	2.1 (1.2)
	With friends	2.1 (1.0)
	With family	1.8 (0.9)

#### Exploratory Factor Analysis

The 4-factor solution retained identified an exergaming for fitness factor (9 items), an exergaming for enjoyment factor (8 items), a factor indicative of preferring exergaming over other gaming options (3 items), and a factor indicative of choosing exergaming over competing interests (eg, sports; 2 items; Table 2).

**Table 2:** Scores by sex and factor loadings of items retained in each RTEX factor (Study 1, NDIT 2017-2019).

RTEX^a^ factor and items within factors		Score						*P* value for sex difference		Factor loading	
		Total (N=272), mean (SD)		Males (N=130), mean (SD)		Females (n=142), mean (SD)					
**Factor 1: Fitness^b^**											
	I exergame to maintain my weight		1.7 (1.1)		1.9 (1.2)		1.5 (1.0)		.004		0.87
	I exergame to maintain my level of fitness		1.8 (1.2)		2.0 (1.3)		1.6 (1.1)		.03		0.86
	I exergame to be more active		2.2 (1.3)		2.4 (1.4)		2.0 (1.2)		.004		0.70
	I exergame to lose weight		1.8 (1.2)		2.0 (1.3)		1.5 (1.0)		.004		0.83
	I exergame to gain strength		1.6 (1.1)		1.8 (1.2)		1.5 (1.0)		.04		0.89
	I exergame to “bulk up”		1.4 (1.0)		1.3 (0.7)		1.4 (0.8)		.44		0.77
	I exergame to gain flexibility		1.9 (1.1)		1.8 (1.2)		1.5 (1.0)		.07		0.90
	I exergame to gain balance		1.7 (1.1)		1.8 (1.2)		1.6 (1.0)		.07		0.88
**Factor 2: Enjoyment^b^**											
	I like to play exergames		3.2 (1.4)		3.2 (1.4)		3.2 (1.3)		.68		0.73
	I like to play exergames with friends		3.3 (1.4)		3.4 (1.4)		3.2 (1.4)		.37		0.81
	I like to play exergames with my family		2.8 (1.5)		2.6 (1.4)		3.0 (1.5)		.03		0.60
	Exergames are boring to play (reverse coded)		3.5 (1.3)		3.5 (1.3)		3.6 (1.3)		.46		0.50
	I exergame to be social		2.5 (1.5)		2.5 (1.4)		2.5 (1.5)		.85		0.60
	Exergames are exciting to play		2.9 (1.3)		2.8 (1.3)		3.1 (1.2)		.02		0.67
	I exergame just for fun		3.5 (1.4)		3.6 (1.4)		3.4 (1.5)		.20		0.72
	I prefer exergames to watching TV^c^		2.0 (1.1)		1.8 (1.1)		2.1 (1.1)		.07		0.41
	I prefer exergames to being on social media (Facebook, Instagram, Snapchat)		2.3 (1.4)		2.0 (1.2)		2.8 (1.4)		<.001		0.44
**Factor 3: Gaming preference^b^**											
	I prefer exergames to more traditional video games^c^		2.4 (1.3)		1.8 (1.1)		2.8 (1.3)		<.001		0.60
	Exergaming is the only type of video game I like		1.5 (1.1)		1.3 (0.8)		1.8 (1.2)		.001		0.81
	Other types of video games bore me		1.6 (1.1)		1.4 (0.9)		1.9 (1.3)		<.001		0.81
**Factor 4: Competing interests^b^**											
	I prefer to play exergames more than outdoor sports		1.8 (1.1)		1.8 (1.2)		1.8 (1.1)		.87		–0.90
	I prefer to play exergames more than indoor sports		1.9 (1.2)		2.0 (1.2)		1.8 (1.1)		.27		–0.84

^a^Reasons to Exergame.

^b^Continuous variable.

^c^Item was not examined in test-retest analysis.

The mean (SD) for factors 1 to 4 were 1.7 (1.0), 3.3 (1.0), 1.9 (1.0), and 1.8 (1.1), respectively. The Cronbach α for the factors ranged from 0.80 to 0.95, indicating adequate internal consistency. A correlation matrix showed that the 4 factors were moderately correlated, with *r* ranging from 0.20 to 0.40. Sex differences were observed in the fitness and gaming preference factors. Females scored higher on both factors, indicating that these factors were more important or true for them as reasons to exergame (Table 3).

**Table 3:** Scores by sex and internal reliability coefficient (Cronbach α) of RTEX factors (Study 1, 2017-2019) and intraclass correlation coefficient for test-retest reliability of RTEX factors (Study 2, 2017).

RTEX^a^ factor		NDIT^b^ sample (N=627), n (%)	Study 1 (n=272)					Study 2 (n=147), ICC^c^ 95% CI
			Mean (SD)	*P* value for sex difference	Range	Cronbach α		
**Factor 1: Fitness**				.02	1.0-4.5	0.95	0.8 (0.6-0.8)	
	Total	265 (42.3)	1.7 (1.0)					
	Male	125 (20.0)	1.6 (0.9)					
	Female	140 (22.3)	1.9 (1.0)					
**Factor 2: Enjoyment value**				.37	1.0-4.8	0.85	0.7 (0.4-0.6)	
	Total	268 (42.7)	2.9 (0.9)					
	Male	127 (20.3)	3.0 (0.9)					
	Female	141 (22.5)	2.9 (0.9)					
**Factor 3: Gaming preference**				<.001	1.0-5.0	0.80	0.8 (0.5-0.7)	
	Total	268 (42.7)	1.9 (1.0)					
	Male	127 (20.3)	1.5 (0.7)					
	Female	141 (22.5)	2.2 (1.1)					
**Factor 4: Competing interests**				.62	1.0-5.0	0.90	0.7 (0.6-0.8)	
	Total	272 (43.4)	1.8 (1.1)					
	Male	130 (20.7)	1.8 (1.0)					
	Female	142 (22.6)	1.8 (1.1)					

^a^Reasons to Exergame.

^b^Nicotine dependence in the teen study.

^c^ICC: intraclass correlation coefficient.

#### Convergent Validity

Correlations between the four RTEX factors and exergaming behavior are presented in Table 4. Multiple linear regression analyses indicated that factor 1 (fitness) was related to exergaming intensity (β= 0.2, 95% CI 0.03-0.4), frequency exergaming alone (β=0.3, 95% CI 0.1-0.5), and frequency exergaming with friends (β=–0.2, 95% CI –0.3 to –0.05). Factor 2 (enjoyment) was related to frequency exergaming alone (β=0.3, 95% CI 0.1-0.5), frequency exergaming with friends (β=0.7, 95% CI 0.6-0.8) and frequency exergaming with family (β=0.5, 95% CI 0.3-0.6). Factors 3 and 4 were not related to exergaming behavior.

**Table 4:** Correlations among variables used in the regression models in Study 1, NDIT 2017-2019.

Variable					Factor 1: Fitness	Factor 2: Enjoyment	Factor 3: Gaming preference	Factor 4: Competing interests	Minutes exergaming/wk	Intensity exergaming	Frequency of exergaming		
											Alone	With friends	With family
**Factor 1: Fitness**													
			*r*		1	0.273^a^	0.312^a^	0.432^a^	0.042	0.325^a^	0.327^a^	0.067	0.162^a^
			*P* value		—^b^	<.001	<.001	<.001	.51	.007	<.001	.28	.009
**Factor 2: Enjoyment**													
		*r*				1	0.227^a^	0.439^a^	0.185^a^	-0.054	0.302^a^	0.625^a^	0.462^a^
		*P* value				—	<.001	<.001	.003	.66	<.001	<.001	<.001
**Factor 3: Gaming preference**													
				*r*			1	0.213^a^	0.062	0.074	0.135^a^	0.165^a^	0.230^a^
				*P* value			—	<.001	.32	.57	.03	.007	<.001
**Factor 4: Competing interests**													
				*r*				1	0.128^c^	0.034	0.285^a^	0.295^a^	0.259^a^
				*P* value				—	.04	.78	<.001	<.001	<.001
**Minutes exergaming/wk**													
				*r*					1	-0.067	0.432^a^	0.145^c^	0.065
				*P* value					—	.56	<.001	.021	.303
**Intensity exergaming**													
				*r*						1	0.301^c^	0.021	0.046
				*P* value						—	.013	.87	.71
**Frequency of exergaming**													
	**Alone**												
				*r*							1	0.180^a^	0.118
				*P* value							—	.003	.056
	**With friends**												
				*r*								1	0.527^a^
				*P* value								—	<.001
	**With family**												
				*r*									1
				*P* value									—

^a^Correlation is significant at the 0.01 level (two-tailed).

^b^Not available.

^c^Correlation is significant at the 0.05 level (two-tailed).

### Study 2

Test-retest reliability coefficients of the measures of exergaming frequency and intensity ranged from 0.4 (for intensity) to 0.9 (for days per week exergaming). The weighted kappa coefficient for ever exergamed was 0.7. Test-retest reliability of RTEX factors ranged from 0.7 to 0.8 (Table 3). Percent or mean of items measuring exergaming behavior by sex is presented in Table 5.

**Table 5:** Items measuring exergaming behavior by sex in Study 1, NDIT 2017-2019.

Exergaming behavior	n	Exergaming behavior items	Study 1			
			Total	Males	Females	*P* value for sex difference
Ever exergamed	627^a^	Have you ever exergamed using a console, cell phone and/or mobile app? % yes	44.4	46.6	42.5	.32
How often – console^a^	257	How often do you exergame using consoles such as Nintendo Wii, XBOX ONE Kinect, Sony Play Station Move, Sony Eye Toy: Kinetic?* (never, <1/month, 1-2/month, 1-3/week, 4-6/week, everyday). Recoded ≥1/week, % yes	17.1	25.2	14.1	.012
How often – mobile^a^	268	How often do you exergame using your cellphone and/or a mobile app? (ex. ZOMBIES, RUN!, Nike+ Running App, Fit Freeway, Pokémon Go)^a^ ((never, <1/month, 1-2/month, 1-3/week, 4-6/week, everyday). Recoded ≥1/week, % yes	34.3	37.7	31.2	.13
Days per week exergaming	62	In the past month, on how many days per week did you exergame using a video game console such as the Nintendo Wii, XBOX 360 Kinect, Sony Play Station Move, Sony Eye Toy: Kinetic? Write 0 if none (open-ended), mean (SD)	0.7 (1.7)	0.9 (2.0)	0.5 (1.5)	.10
Minutes per bout	62	On average how many minutes did you spend each time you did this? Write 0 if none*(open-ended), mean (SD)	44.9 (35.0)	50.2 (39.5)	37.5 (28.6)	.17
Intensity	72	What was your level of effort when you did the activity? (%) Light Moderate Intense	63.0 35.7 1.4	70.0 27.5 2.5	56.3 43.8 0	.26

^a^In NDIT cycle 23 (44% had ever exergamed; n=272) were included in the analysis.

## Discussion

This study describes the development and validation of a new scale that measures general motivation and reasons to exergame. RTEX incorporates 4 factors including exergaming for fitness, exergaming for enjoyment, preferring exergaming over other gaming choices, and choosing exergaming over competing interests (eg, sports). A total of 26 items were considered. Four were eliminated (Multimedia Appendix 1) because, according to theory and statistical criteria, they did not contribute to improving the structure of any factor. Although a key reason for conducting EFA is to eliminate items to increase reliability (precision) and readability of a scale, these four items may be appropriate in other populations and should be further investigated. RTEX factors were internally consistent and had adequate test-retest reliability. Because a primary goal of gaming is to provide motivational affordances (ie, how the game environment invites and maintains motivation) [50], identification of motivational attributes is key to designing games and gamified systems that are sustainable [24].

Two of the four factors identified in RTEX demonstrated convergent validity against exergaming frequency and intensity, which are key outcomes in evaluating exergaming as a method to increase PA and reduce sedentary behavior [51]. Specifically, participants who exergamed for fitness or enjoyment reported a higher frequency of exergaming socially. Individuals who scored higher on the fitness factor may exergame purposely to lose or maintain weight or to bulk up, which are extrinsic motivations according to the self-determination theory [52], suggesting that intrinsic rather than extrinsic motivation relates to intentions to engage in more PA [53]. The sustainability of exergaming for fitness reasons should be further investigated in the context of internal and external motivation and the self-determination theory.

Our finding that the enjoyment factor relates positively to frequency exergaming with friends, family and alone aligns with Self-Determination Theory [54]. The items in factor 2 encompass intrinsic reasons to exergame (ie, fun, competence, relatedness, enjoyment, excitement, preferences, autonomy), suggestive that games, gamified systems, and exergaming interventions should be designed to permit experiences that are intrinsically motivating [54]. This aligns with results of a study on Pokémon, in which the authors reported that the social aspect of the game was one of the strongest predictors of sustained gameplay [55]. Since enjoyment is central to increasing and maintaining motivation for PA generally and exergaming specifically, exergame developers will need to incorporate elements to increase enjoyment or that help increase intrinsic motivation among those who exergame primarily for external fitness reasons [52,53]. For example, games that provide external motivation through ongoing feedback on physical form, effort, and progress in the game may need to also include components that increase enjoyment and autonomy (eg, providing more choices within the game, goal setting). Increasing or focusing on intrinsic motivation to encourage sustainable use among those who exergame for fitness may help increase exergaming sustainability [56], and assessing an individual’s motivation for exergaming will be key when prescribing exergaming in an intervention or by health practitioners [57].

Preferring exergaming over other gaming options and choosing exergaming over competing interests (eg, sports) were not related to exergaming frequency or intensity. The items in these two factors address elements that inform overall motivations and reasons to choose exergaming over traditional video games or sports, which may not be associated with exergaming behavior, at least as we have measured it. Alternatively, preferences may not translate into exergaming behavior.

Sex differences were apparent in the fitness and gaming preference factors. Specifically, compared to males, females preferred exergames over traditional video games and scored higher on the fitness factor. Consistent with previous work [58-60], females may exergame to incorporate PA into their lives, whereas males enjoy gaming, in general, as a pastime, and play for fun. If replicated, exergaming interventions may need to incorporate different “prescriptions” for males and females.

Our measures of exergaming frequency and intensity, which were modelled after the International Physical Activity Questionnaire [42], have been used in previous studies [58-60], but their test-retest reliability was unknown. In this study, retest reliability was adequate. Given the popularity of exergaming, these items (with the exception exergaming intensity, which requires further work) could be considered for use in observational and interventional studies as well as in surveillance systems.

One limitation of this study is that self-report data are subject to misclassification. In addition, our findings are limited to young adults, although exergaming may benefit all ages. Finally, we did not distinguish between mobile exergaming, which has increased in popularity, and console gaming.

### Conclusion

In this study, we introduce the RTEX, which provides a valid and reliable assessment of reasons to exergame and therefore has promise in terms of increasing the evidence that informs sustainable exergaming. Future research should examine the psychometric properties of the RTEX in diverse samples including children and older adults and should measure exergaming behavior objectively using feedback from exergaming consoles or accelerometers. Our data suggest that there are sex differences in RTEX, a tenet that requires further exploration. Finally, whether the RTEX relates to other health outcomes and behaviors should be investigated.

## Appendix

Multimedia Appendix 1 Items removed from among original RTEX items, based on psychometric analyses.

## Abbreviations

EFA: exploratory factor analysis
ICC: intraclass correlation coefficient
NDIT: nicotine dependence in teens study
PA: physical activity
RTEX: reasons to exergame scale
