# Supplementary material for: Development and Validation of the Reasons to Exergame (RTEX) Scale in Young Adults: Exploratory Factors Analysis
Source: JMIR Serious Games. 2020 Jun 15;8(2):e16261. doi: 10.2196/16261 (PMC7325003; doi:10.2196/16261)
Supplement: Multimedia Appendix 1 [file games_v8i2e16261_app1.docx]

| Multimedia Appendix 1: Items removed from among original RTEX items, based on psychometric analyses |
| --- |
| Eliminated items |
| Exergames are irritating to play |
| I think that playing exergames is a good way to integrate physical activity into my life |
| Exergames are calming to play |
| I think that I will play exergames for many years |
